# Energy transfer between the nicotinamide nucleotide transhydrogenase and ATP synthase of *Escherichia coli*

**DOI:** 10.1038/s41598-021-00651-6

**Published:** 2021-10-27

**Authors:** Simone Sandra Graf, Sangjin Hong, Philipp Müller, Robert Gennis, Christoph von Ballmoos

**Affiliations:** 1 Department of Chemistry, Biochemistry & Pharmaceutical Sciences, University of Bern, Freiestrasse 3, 3012 Bern, Switzerland; 2 Department of Biochemistry, University of Illinois at Urbana-Champaign, Urbana, IL 61801 USA

**Keywords:** Biochemistry, Biological techniques, Bioenergetics, Ion transport

## Abstract

Membrane bound nicotinamide nucleotide transhydrogenase (TH) catalyses the hydride transfer from NADH to NADP^+^. Under physiological conditions, this reaction is endergonic and must be energized by the *pmf*, coupled to transmembrane proton transport. Recent structures of transhydrogenase holoenzymes suggest new mechanistic details, how the long-distance coupling between hydride transfer in the peripheral nucleotide binding sites and the membrane-localized proton transfer occurs that now must be tested experimentally. Here, we provide protocols for the efficient expression and purification of the *Escherichia coli* transhydrogenase and its reconstitution into liposomes, alone or together with the *Escherichia coli* F_1_F_0_ ATP synthase. We show that *E. coli* transhydrogenase is a reversible enzyme that can also work as a NADPH-driven proton pump. In liposomes containing both enzymes, NADPH driven H^+^-transport by TH is sufficient to instantly fuel ATP synthesis, which adds TH to the pool of *pmf* generating enzymes. If the same liposomes are energized with ATP, NADPH production by TH is stimulated > sixfold both by a pH gradient or a membrane potential. The presented protocols and results reinforce the tight coupling between hydride transfer in the peripheral nucleotide binding sites and transmembrane proton transport and provide powerful tools to investigate their coupling mechanism.

## Introduction

NADH and NADPH and their oxidized counterparts NAD^+^ and NADP^+^ are important cofactors in countless cellular reactions. While NADH is formed during catabolism by many different enzymes and consumed mainly during oxidative phosphorylation, NADPH is majorly regenerated by only a handful of cellular systems found in the cytosol or the mitochondria^1^, their individual contribution depending on the respective tissue^2^, but also the growth conditions^3^. However, it should be noted that there are indications that also other NADPH generating reactions seem to play a role in maintaining the NADPH/NADP^+^ redox balance^3^. A unique system is the proton pumping transhydrogenase (TH) in the mitochondrial inner membrane or the bacterial cytoplasmic membrane which couples the reduction of NADP^+^ to the oxidation of NADH via direct hydride transfer according to the following equation:

1 $$NADH+ {NADP}^{+}+ {H}_{out}^{+} \leftrightarrow {NAD}^{+}+NADPH+ {H}_{in}^{+}$$

with “out” meaning the periplasmic space and “in” meaning the cytosol. Although membrane bound TH is not essential in *E. coli*, it appears to be the major source of NADPH in contrast to most other investigated microbes that rely majorly on the oxidative pentose phosphate pathway and the isocitrate dehydrogenase for NADPH generation^4^. Under physiological conditions, the TH forward reaction is endergonic and requires energization by the *pmf*^5^. Therefore, TH employs transmembrane proton translocation to energize hydride transfer between NADH and NADP^+^.

**Figure 1:**
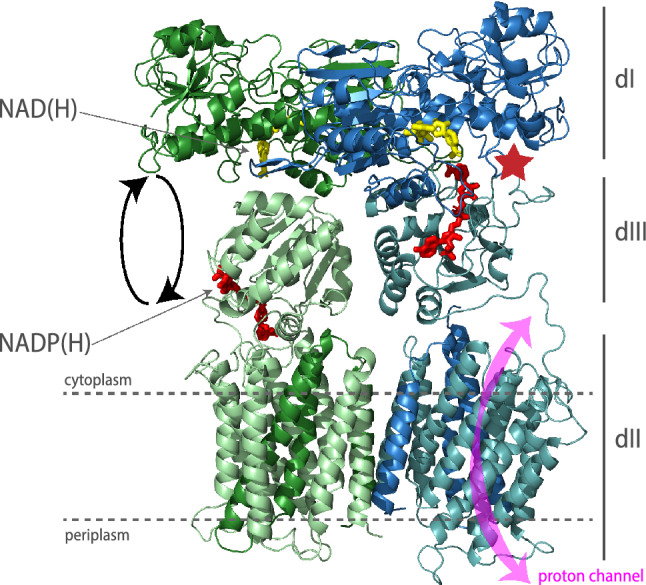
Homology model of *E. coli* transhydrogenase. Representation of the homology model of *E. coli* transhydrogenase based on the crystal structure of *Thermus thermophilus* transhydrogenase. The protein is shown as the homodimer as which it is present in the membrane. The two monomers are coloured blue and green respectively. Each monomer is composed of two subunits (lighter and darker shade of green and blue, respectively) which form three domains. For instance in the green monomer, domain I (dI) and part of domain II (dII) are formed by subunit α(dark green), whereas the remaining of dII and domain III (dIII) are formed by subunit β (light green). Shown in red and yellow are NADP(H) and NAD(H) respectively at their respective binding sites. dIII is in an asymmetric orientation in the two monomers with one dIII facing upwards and one dIII facing downwards. The NADP(H) binding domain dIII is believed to flip up and down by about 180° in order to bring the NADP(H) binding site close to the NAD(H) binding site or the proton channel. Hydride transfer (indicated by red star) happens when dIII is facing upwards. The proton channel is located in dII (indicated by pink double headed arrow). Structure rendered in Pymol (Schrödinger, v2.4.0, open- source version) and labelled in Adobe Illustrator 2021.

The reaction is completely reversible and can thus also occur in the reverse direction, converting TH to a redox-driven proton pump without redox cofactor^6^. In mitochondria and bacteria, the enzyme exists as a functional dimer with each protomer containing three structural domains (Fig. 1). The hydrophilic domains dI and dIII protrude into the cytoplasmic space and bind NAD(H) (Fig. 1, yellow) and NADP(H) (Fig. 1, red) respectively, whereas dII is the transmembrane portion of the enzyme^7^. Hydride transfer happens at the dI/dIII interface (Fig. 1, red star) and is coupled to proton translocation across the membrane in dII (Fig. 1, pink double headed arrow). Depending on the species, TH consists of one, two, or three polypeptide chains as for example in humans, *E. coli,* and *Thermus thermophilus,* respectively^7^. The two subunits of the *E. coli* enzyme, α and β, have a molecular mass of 54 and 48 kDa, respectively^8^.

Research on nicotinamide nucleotide transhydrogenase already started in the 1950-ies by Colowick and Kaplan^9–12^. It was suggested that transhydrogenase was controlled by ATP^13,14^ and later found that both NADH and ATP increased the rate of NADPH production by transhydrogenase in submitochondrial particles^15,16^. Transhydrogenase was thus the first enzyme being demonstrated to use the energy derived from the respiratory chain without being involved in the phosphorylation machinery. Purified transhydrogenase from beef heart mitochondria was extensively studied in reconstitution experiments by Earle and Fisher^17^ or Enander and Rydström^18^, including coreconstitution of transhydrogenase with ATP synthase from bovine heart^19,20^. Since an ATP hydrolysis generated *pmf* accelerated the TH forward reaction considerably, the coupling between *pmf* and hydride transfer was recognized to be mediated by the transport of one proton from the *P*- to the *N*-side per every hydride being transferred to form NADPH^19–22^.

Isolation of the *E. coli* transhydrogenase gene by Clarke and Bragg in 1985^23^ opened the way for mutational studies and shifted the focus to the *E. coli* enzyme. For a conclusive summary of investigated mutant variants see^24^. Importantly, residue βH91 in domain II, which is the only conserved charged residue in the putative proton channel, was identified as key amino acid involved in proton translocation^8,25–30^. Other important residues related to proton translocation are βN222, βD213, βC260-βS266, but their exact role remained unclear^28,30–32^. As TH does not contain any redox factors which could couple electron transport to proton translocation, a mechanism driven by conformational changes was envisaged^33^. Structural information on the soluble domains dI^34,35^ and dIII^36,37^, as well as the dI_2_-dIII trimer^38,39^ triggered a series of structure based mutagenesis studies investigating hydride transfer^33^. In 2015, finally, the first crystal structure of the holoenzyme from *T. thermophilus* in the presence of NADP^+^ at low resolution and the isolated dII domain at high resolution were published^6^. In the structure, the two NADP(H) binding domains dIII were in asymmetrical orientation, one copy being positioned “face-up” with its NADP(H) binding site in close proximity to the NAD(H) binding site in dI, and the other copy oriented “face-down”^6^. It was suggested that dIII swivels up and down bringing the NADP nucleotide close to either NAD^+^ or the proton channel, coupling proton pumping to hydride transfer. For a more detailed description see^6,7^. In 2019, the mechanism was fine-tuned thanks to high resolution cryo-EM structures of the mitochondrial TH from *Ovis aries* in the presence and absence of nucleotides^24^. In summary, the new mechanism states that the redox state of NADP controls protonation of the critical histidine (βH91 in *E. coli*) in the channel by influencing its pKa and that conformational changes within the channel are directly linked to dIII attachment or detachment from dII^24^. Both models are based on structures of solubilized enzymes and require experimental verification, especially the proposed interaction between the face-down dIII domain and the presumable proton loading site histidine in the membrane. It is further unclear in which step the *pmf*, consisting of a pH gradient and the membrane potential Δψ, affects the molecular mechanism. The investigation of the *pmf* dependent coupling mechanism requires the presence of a tight membrane and the protein inserted in this membrane. Critical amino acid residues are investigated using mutant variants that requires a robust experimental system allowing convenient genetic manipulation and production of the enzyme. In the following, we describe the construction, over-expression and purification of *E. coli* transhydrogenase, as well as a basic functional characterization based on functional assays in solution. In addition, we provide protocols for the straight-forward reconstitution of TH alone and in presence of *E. coli* ATP synthase. We show for the first time that in liposomes containing both enzymes, TH generates a sufficiently large *pmf* to energize ATP synthesis by the ATP synthase. While some of these experiments have been described before^17–20^, we are convinced that our modern straight-forward experimental setup is timely to help researchers elucidating the enigmatic molecular mechanism of energy coupled transhydrogenase.

## Materials and methods

If not stated otherwise, chemicals were purchased at Merck-Sigma-Aldrich.

### Generation of *E. coli* transhydrogenase overexpression strain

The *E. coli* transhydrogenase *pnt* genes were subcloned from pSA2 carrying *pntAB* with a His-tag^40^ into pET16b using *Nco*I and *Bam*HI sites to generate pET16b-EcTH. The pET16b-EcTH was used to transform *pntAB* knockout strain (THO, C43(DE3)Δ*cyo*Δ*pntAB*) in which *pnt* genes were replaced by chloramphenicol resistance gene. The transformed cells were plated onto LB plates containing 30 µg/ml chloramphenicol and 100 µg/ml ampicillin. The transformants grown overnight at 37 °C were selected and the pET16b-EcTH from the selected transformants was sequenced to check the full DNA sequence of *pntAB*.

### Expression and purification of enzymes

C43 ∆*cyo* ∆*pntAB* cells were transformed with pET16b plasmid carrying the WT *E. coli* transhydrogenase. Using primers (5’-CGAGGCTAGCATGCGAATTGGCATACCAAG-3’ and 5’-CAGCCGGATCCTTACAGAGCTTTCAG-3’), *E. coli pnt* operon was amplified from genomic DNA, cut with *Nhe*I/*Bam*HI and ligated to the *Nco*I/NheI-digested fragment of pET28a that includes a His-tag and a thrombin site. The ligation product was ligated into pET16b linearized with *Nco*I/*Bam*HI. The presence of *pnt* genes and a His-tag were confirmed by DNA sequencing. The final plasmid codes for subunits α and β, and a hexa-His-Tag at the N-terminus of subunit α. Transformed cells were plated onto LB agar plates containing 50 μg/ml kanamycin and 200 μg/ml ampicillin (kana^50^/amp^200^) and incubated at 37 °C overnight. A single colony was inoculated into 5 ml LB (kana^50^/amp^200^) and incubated at 37 °C and later supplemented with 50 ml LB (kana^50^/amp^200^) and grown overnight. The next day, precultures were used to inoculate expression cultures (50 ml per liter) and incubated at 37 °C. When OD_600_ reached ~ 0.5 to 0.7, protein expression was induced by addition of 1 mM isopropyl-β-d-thiogalactopyranoside (IPTG) with subsequent expression overnight at 30 °C. The next morning cells were harvested (5000×*g* for 20 min at 4 °C) and the cell pellets were resuspended in 50 mM MOPS, pH 7.0, 100 mM KCl. A spatula tip of each, Pefabloc®, phenylmethylsulfonylfluoride (PMSF) and DNase I were added and the cells were disrupted by three passages through a Maximator HPL6 (Maximator AG, Switzerland). Unbroken cells and cell debris were collected by a low spin at 20,000×*g* for 20 min at 4 °C followed by membrane collection in a high spin at 180,000×*g* for 2 h at 4 °C. The membranes were resuspended in 50 mM MOPS, pH 7.8 and the total protein concentration was determined by BCA using BSA as a standard (Pierce™ BCA Protein Assay Kit). Protein concentration was adjusted to 5 mg/ml by dilution, supplemented with 10 mM imidazole and 1% n-dodecyl β-d-maltoside (DDM) and membranes were solubilized for 2 h at 4 °C followed by ultracentrifugation to collect unsolubilized material (180,000×g for 20 min at 4 °C). The solubilisate was loaded onto a prepacked 5 ml HisTrap FF column (GE-healthcare) at 4 °C using a peristaltic pump. The column was washed with 20 column volumes (CV) of 50 mM phosphate buffer pH 7.8, 20 mM imidazole, 0.02% DDM, and TH was eluted with 50 mM phosphate buffer pH 7.8, 240 mM imidazole, 0.02% DDM. Two millilitre fractions were collected and fractions containing TH were pooled and concentrated in an Amicon® Ultra 15 ml filter with 100 kDa cutoff. The buffer was exchanged to 50 mM phosphate buffer pH 7.8, 0.1% DDM. Protein concentration was determined using Coomassie stained SDS-PAGE with BSA as a standard and 10% glycerol was added to the purified protein prior to freezing it as droplets in liquid nitrogen and storage at − 80 °C. Repeated thawing and freezing of the enzyme was omitted due to loss of activity.

### Activity measurements with detergent-solubilized enzyme

Forward activity was measured in 50 mM HEPES pH 7.5, 0.1% DDM under nucleotide regenerating conditions as described^41^. To determine the K_M_ of NADH in the forward reaction, NADP^+^ was held constant using glutathione reductase and oxidized glutathione (0.5 mM NADP^+^, 4 U/ml glutathione reductase, 1 mM oxidized glutathione and various concentrations of NADH) whereas determination of K_M_ for NADP^+^ was obtained using alcohol dehydrogenase to hold NADH concentrations constant (50 μM NADH, 1.2 U/ml alcohol dehydrogenase, 130 mM ethanol, 6 mM hydrazine (pH 7.0) and various concentrations of NADP^+^). See Supplementary Fig. S1.

Reverse activity was measured in 50 mM HEPES pH 7.5, 0.1% DDM under nucleotide regenerating conditions. K_M_ of NADPH was determined by regenerating NAD^+^ using lactate dehydrogenase and pyruvate (0.5 mM NAD^+^, 180 mU/ml lactate dehydrogenase, 3.3 mM pyruvate and various concentrations of NADPH). K_M_ of NAD^+^ was determined using glucose-6-phosphate dehydrogenase and D-glucose-6-phosphate to regenerate NADPH (100 μM NADPH, 200 mU/ml glucose-6-phospohate dehydrogenase, 5 mM MgCl_2_, 2 mM D-glucose-6-phosphate and various concentrations of NAD^+^). See supplementary Fig. S1.

### Reconstitution of transhydrogenase into liposomes

Soybean lecithin (90% PC content, Alfa Aesar) was resuspended in 50 mM HEPES, pH 7.5 100 mM KCl at 5 mg/ml, followed by seven cycles of flash freezing in liquid nitrogen and thawing at 29.4 °C and extruded 21 × through a 200 nm membrane (Whatman® Nuclepore Track-Etched Membrane). Liposomes were destabilized by the addition of octyl β-D-glucopyranoside (OG) to a final concentration of 0.6% followed by addition of 28 μl of purified *E. coli* transhydrogenase (108 µM) to 1 ml of liposomes and incubation at RT for 15 min on a rotating wheel. The detergent was removed by addition of BioBeads SM^2^ (BioRad) to a final concentration of 400 mg/ml and incubation at 4 °C on a rotating wheel for 4 h. Before measurements, the proteoliposomes were mixed 1:1 with the same buffer and kept on ice.

Orientation of reconstituted TH in proteoliposomes was determined using alamethicin which forms a membrane pore allowing NADH to pass through and thereby also reach nucleotide binding sites located on the inside of the proteoliposome. Reverse and cyclic activity of proteoliposomes containing transhydrogenase were determined as described above. Briefly, reverse activity was measured in 1.5 ml 50 mM HEPES, pH 7.5, 100 mM KCl, containing 50 μl proteoliposomes, 1 μM CCCP, 0.2 μM valinomycin, 100 μM NADPH and in presence or absence of 6.7 μg/ml alamethicin. The reaction was started with the addition of 200 μM APAD^+^ and progress of the reaction was monitored at 375 nm. Cyclic activity was measured in 1.5 ml 50 mM HEPES, pH 7.5, 100 mM KCl, containing 50 μl proteoliposomes, 1 μM CCCP, 0.2 μM valinomycin, 200 μM NADH, 10 μM NADP^+^ and in presence or absence of 6.7 μg/ml alamethicin. The reaction was started with the addition of 200 μM APAD^+^ and progress of the reaction was monitored at 375 nm.

### NADPH driven proton pumping

Proton pumping during reverse activity of transhydrogenase was measured following ACMA (9-Amino-6-Chloro-2-Methoxyacridine) fluorescence at λ_ex/em_ 419/483 nm in a PTI Quantamaster. For each measurement, 100 μl of liposomes were resuspended in 1.5 ml buffer containing 50 mM HEPES pH 7.5, 100 mM KCl, 180 mU/ml lactate dehydrogenase, 2 μM ACMA, 0.2 μM valinomycin. Depending on the starting conditions, 200 μM NADPH or 500 μM NAD^+^ were also present or added to start the reaction after recording a baseline. The proton gradient was dissipated by addition of NH_4_Cl to a final concentration of 33 mM.

### NADH driven proton pumping

Transmembrane proton pumping during the forward reaction was measured by following membrane potential generation using TMRE fluorescence at λ_ex/em_ 549/573 nm in a PTI Quantamaster in 1.5 ml 50 mM HEPES pH 7.5, 100 mM KCl, 50 μl transhydrogenase proteoliposomes, 4 U/ml glutathione reductase, 1 mM oxidized glutathione, 0.2 μM TMRE and 200 μM NADH. The reaction was started by addition of NADP^+^ (final concentration 800 μM).

### Co-reconstitution of transhydrogenase and ATP synthase

ATP synthase was purified as described^42^. Liposomes were prepared as above and destabilized by addition of 0.6% OG, followed by addition of 28 μl of *E. coli* transhydrogenase (108 µM) and 28 µl of *E. coli* ATP synthase (44 μM) per ml liposome. Proteoliposomes were incubated at RT for 15 min on a rotating wheel. Subsequently, BioBeads (SM^2^, BioRad) were added to a final concentration of 400 mg/ml and the sample was incubated at 4 °C on a rotating wheel for 4 h. Before measuring the proteoliposomes were mixed 1:1 with reconstitution buffer (50 HEPES, pH 7.5, 100 mM KCl).

### Transhydrogenase forward reaction in the co-reconstituted system

#### ACMA dequenching assay

ACMA dequenching was measured using proteoliposomes containing *E. coli* transhydrogenase and ATP synthase. Measurements were done in 1.5 ml 50 mM HEPES, pH 7.5, 100 mM KCl, 100 μl liposomes, 0.2 µM valinomycin, 4 mU/ml glutathione reductase, 1 mM oxidized glutathione, 2 µM ACMA, 500 µM NADP^+^, and 2 mM MgCl_2_. After recording the baseline, an acidic inside proton gradient was established by addition of 250 µM ATP. After a stable plateau was reached, transhydrogenase was switched on in the forward direction by addition of NADH to a final concentration of 500 µM. The proton gradient was dissipated by addition of 33 mM NH_4_Cl.

#### Transhydrogenase driven ATP synthesis

ATP synthesis using proteoliposomes containing *E. coli* transhydrogenase and ATP synthase was measured using a Glomax Luminometer (Promega). For each measurement, 100 μl proteoliposomes were suspended in 400 µl 50 mM HEPES, pH 7.5, 100 mM KCl, 5 mM MgCl_2_, 40 mM KH_2_PO_4_, 0.4 mg/ml CLS II luciferase kit reagent (Roche), 250 µM ADP, and 1.5 mM NAD^+^. After recording basal ATP levels, the reaction was started by addition of 750 µM NADPH.

### ATP hydrolysis stimulated transhydrogenase forward activity

The forward activity in presence and absence of a membrane potential established by ATP synthase was measured in 1.5 ml 50 mM HEPES, pH 7.5, 100 mM KCl containing 1.2 U/ml alcohol dehydrogenase, 130 mM ethanol, 6 mM hydrazine (pH 7.0), 100 µl liposomes, 4 mM MgCl_2_, and 200 µM NADH in presence and in absence of 0.2 µM valinomycin as well as in presence of 0.2 μM valinomycin and 1 μM CCCP. The reaction was started by addition of 500 µM NADP^+^ and NADPH formation was followed measuring absorbance at 340 nm on a Cary 60 spectrophotometer. After recording the signal for approximately 200 s, ATP synthase was switched on by adding 250 µM ATP.

### *pmf* induced switch from reverse to forward transhydrogenase activity

Using liposomes containing ATP synthase and transhydrogenase, the measurements were performed in 1.5 ml 50 mM HEPES, pH 7.5, 100 mM KCl containing 100 μl liposomes along with 4 mM MgCl_2_, and 150 µM NADPH. First, the transhydrogenase reaction was started in reverse direction by addition of 150 µM APAD^+^. After a plateau was reached, indicating chemical equilibrium, 250 µM ATP was added. The reaction was followed by measuring absorbance at 375 nm to monitor reduction of APAD^+^ on a Cary UV/Vis spectrophotometer.

## Results

### Cloning and purification

**Figure 2:**
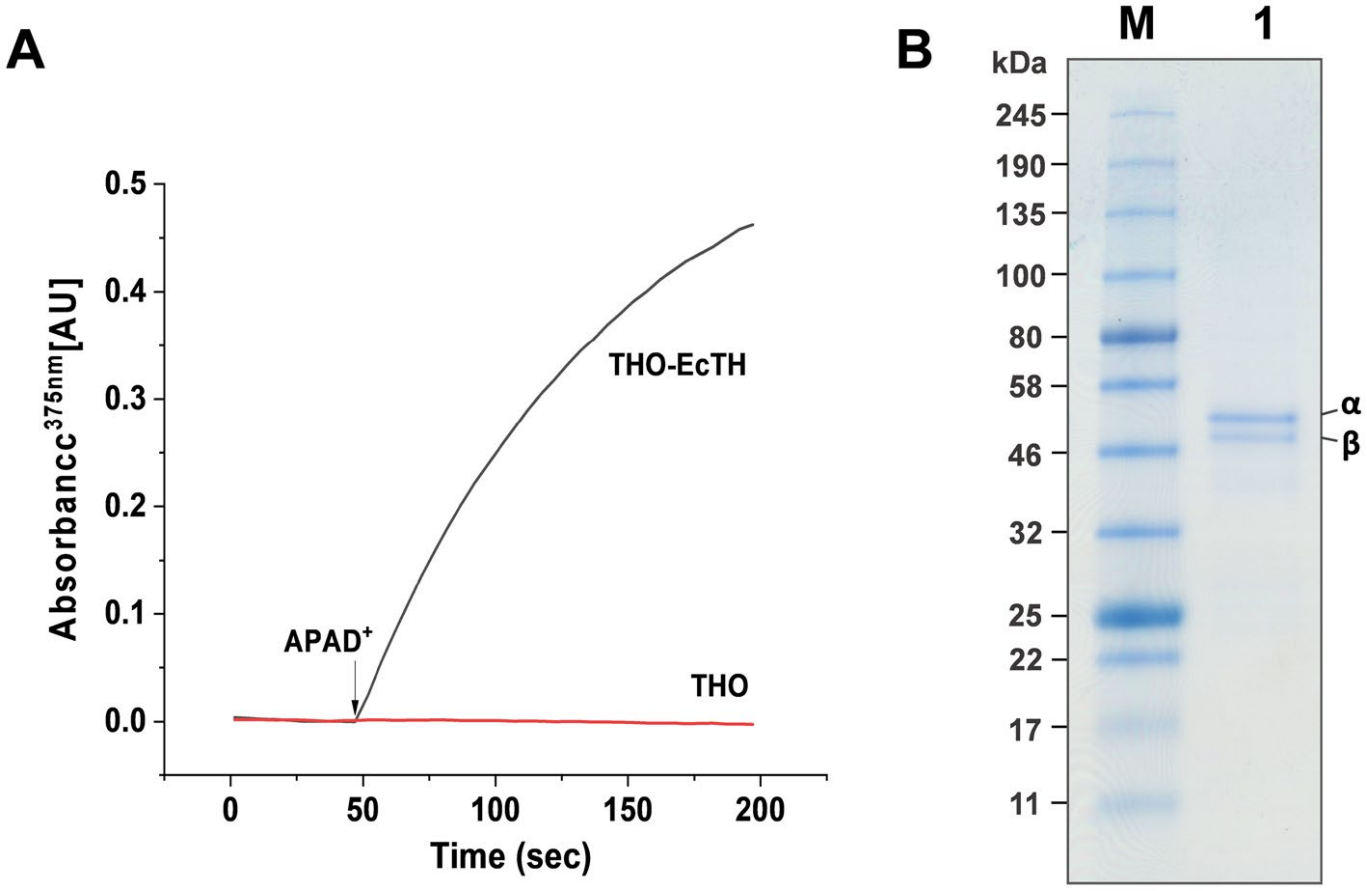
Overexpression and purification of *E. coli* transhydrogenase. (**A**) Reverse transhydrogenase activity of *E. coli* transhydrogenase in native membranes was measured spectrophotometrically by following the reduction of APAD^+^ by NADPH at 375 nm. The reactions were carried out in 0.8 ml of 50 mM MOPS, pH 7.0 containing 100 mM KCl, 200 µM NADPH and 6 µM CCCP, and the reaction was started by adding 400 µM APAD^+^. The data were normalized to the total protein concentrations in the membranes. THO and THO-EcTH represent the membranes from THO and THO with *E. coli* transhydrogenase expressed from pET16b-EcTH, respectively. (**B**) The SDS-PAGE was conducted on 4–20% precast PAGE gel with Coomassie brilliant blue staining. Prestained protein marker, 11–245 kDa from New England BioLabs (lane M) was used. Approximately 1.2 μg of purified protein was loaded (lane 1). Subunits α (57.1 kDa with a Hexahis-tag) and β (48.7 kDa) are indicated. Graph created in Prism software (GraphPad, v7.0) and gel was labelled in Adobe Illustrator 2021.

The *pntAB* operon for *E. coli* transhydrogenase was subcloned into a pET16b vector, placing the genes under the control of the IPTG-inducible T7 promoter, yielding pET16b-EcTH. The plasmid was transformed into a transhydrogenase knockout *E. coli* strain (THO), and the protein was overexpressed following IPTG induction. The expression of *E. coli* transhydrogenase exhibited no significant toxicity, and the yield of purified protein was 5–6 mg/L. Protein expression was verified in native membranes (Fig. 2A), followed by purification using NTA-affinity chromatography. After a single purification step, the protein was essentially pure as judged by SDS-PAGE and was used for functional experiments without further purification (Fig. 2B). The detergent solubilized protein showed reverse, forward and cyclic activity. In contrast to forward and reverse activity, cyclic activity is not accompanied by proton transport, but the hydride is transferred from NADH over NADP^+^ back to NAD^+^. Specific activity during the reverse reaction of the purified detergent solubilized enzyme was 5.4 ± 1 μmol/mg/min (n = 7).

### Activity measurements and K_M_ determination using detergent solubilized enzyme

In a first set of experiments, we determined the apparent K_M_ of NADPH and NAD^+^ during the reverse reaction under Michaelis–Menten conditions according to the following equation:

$${NAD}^{+}+NADPH+ {H}_{in}^{+} \to NADH+ {NADP}^{+}+ {H}_{out}^{+}$$

To this end, NAD^+^ was held constant at 0.5 mM using lactate dehydrogenase and pyruvate, and the K_M_ of NADPH was determined measuring catalytic activity at different NADPH concentrations. For the K_M_ of NAD^+^, different NAD^+^ concentrations were added in the presence of 100 μM NADPH which was held constant using glucose-6-phosphate dehydrogenase and D-glucose-6-phosphate as a regenerating system (Fig. S1).

Next, we determined the apparent K_M_ values of substrates NADH and NADP^+^ during the forward reaction according to the following equation:

$$NADH+ {NADP}^{+}+ {H}_{out}^{+}\to {NAD}^{+}+NADPH+ {H}_{in}^{+}$$

To determine the K_M_ of NADH, NADP^+^ was held constant at 0.5 mM using glutathione reductase and oxidized glutathione while various concentrations of NADH were added. Determination of K_M_ for NADP^+^, on the other hand, was obtained using alcohol dehydrogenase to hold NADH concentration constant at 500 μM while adding various concentrations of NADP^+^ (Fig. S1). In all measurements, the activity was followed over time measuring absorption at 340 nm where both NADH and NADPH show an absorption maximum. The K_M_ values determined from these experiments are summarized in Table 1.

**Table 1:** K_M_ values determined for all substrates in the reverse and forward reactions.

Reverse reaction	K_M_ NADPH [μM] 4.1 ± 1.2	K_M_ NAD^+^ [μM] 108 ± 12
Forward reaction	K_M_ NADP^+^ [μM] 37 ± 7	K_M_ NADH [μM] 6.8 ± 1

The respective initial reaction velocities were analysed in a double reciprocal plot with the corresponding nucleotide concentration (Lineweaver–Burk Plot). Shown are averaged values and standard deviations from measurements of three enzyme preparations.

### Reconstitution of purified TH into preformed liposomes

**Figure 3:**
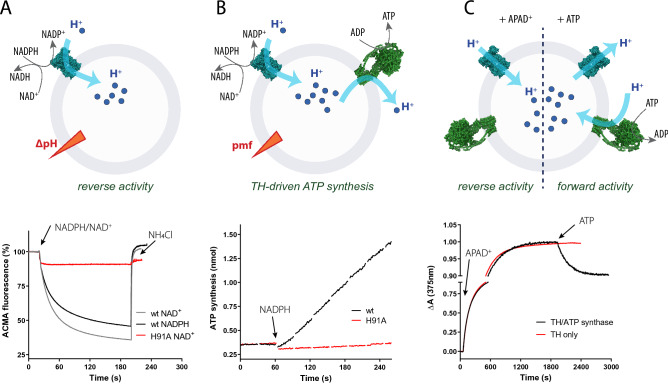
Reverse activity of EcTH in reconstituted systems. (**A**) Proton pumping of reconstituted transhydrogenase during reverse activity was measured following ACMA fluorescence in 1.5 ml 50 mM HEPES pH 7.5, 100 mM KCl, supplied with 100 μl transhydrogenase proteoliposomes, 180 mU/ml lactate dehydrogenase, 0.2 μM valinomycin, and 200 μM NADPH. Upon addition of 500 μM NAD^+^ or NADPH, ACMA fluorescence decreased until a plateau was reached. The proton gradient was then disrupted by addition of 33 mM NH_4_Cl leading to recovery of ACMA fluorescence. (**B**) Transhydrogenase-driven ATP synthesis was measured using co-reconstituted ATP synthase and TH proteoliposomes in 50 mM HEPES, pH 7.5, 100 mM KCl, containing 100 μl liposomes along with 5 mM MgCl_2_, 40 mM KH_2_PO_4_, 0.4 mg/ml CLS II luciferase kit reagent, 250 µM ADP, and 1.5 mM NAD^+^. The reaction was started by addition of 750 µM NADPH leading to ATP synthesis which can be followed as a linear increase of the luminescence signal. **C)** Switch of TH directionality upon *pmf* formation by ATP synthase. In 1.5 ml buffer (50 mM HEPES, pH 7.5, 100 mM KCl, 4 mM MgCl_2_, 150 μM NADPH) and 100 μl proteoliposomes containing co-reconstituted ATP synthase and transhydrogenase were suspended. First, transhydrogenase reverse activity was started by adding 150 µM APAD^+^ which led to an increase in absorbance at 375 nm representing formation of APADH. Turning on ATP synthase by adding 250 µM ATP was followed by a decrease in absorbance at 375 nm, indicating that APADH was oxidized and transhydrogenase activity was switched to the forward direction. Cartoon schemes were drawn in Adobe Illustrator 2021 and graphs created in Prism software (GraphPad, v7.0).

As described above for the solubilized enzyme, the hydride transfer reaction catalysed by TH is fully reversible and the direction of the reaction is dictated by the relative concentrations of the present substrates. The same is true for the membrane embedded enzyme, but in addition to hydride transfer, proton translocation across the membrane takes place in either direction, depending on the directionality of the reaction. To investigate the coupling of hydride transfer to proton translocation, the enzyme must be embedded into tight vesicles. We prepared soybean lecithin liposomes and incorporated TH via detergent-mediated reconstitution. Trying several reconstitution approaches^43^, we were most successful using 0.6% octyl glucoside to destabilize preformed liposomes and Bio-Beads to remove the detergent after a short incubation of the enzyme with the destabilized liposomes. Other detergents (DDM or cholate for instance) or methods for detergent removal (gel filtration or cyclodextrin) yielded proteoliposomes with lower proton pumping activities. Success of reconstitution was tested monitoring NADPH-driven proton pumping using an ACMA quench assay (Fig. 3A). Reconstitution efficiency of transhydrogenase was estimated using SDS-PAGE, visualizing the steps of the reconstitution process (soluble enzyme, low spin supernatant, high spin supernatant and pellet). Following this approach, an incorporation efficiency of 65 ± 10% is estimated. Orientation of the reconstituted TH was estimated comparing activities of outwards oriented enzymes and total enzyme. To this end, reverse TH activity was measured in the absence and in presence of the membrane pore-forming peptide alamethicin that allows the membrane impermeable nucleotides to reach the binding sites of inwards-oriented TH. The same experiment was also performed following cyclic activity (Fig. S2). These measurements revealed a preferential orientation of TH with the nucleotide binding sites situated on the outside of the liposomes (75 ± 5% using reverse activity, 85 ± 5% using cyclic activity).

### NADPH-driven *pmf* generation and ATP synthesis

We start with the description of experiments where liposome-reconstituted TH was run in the reverse direction, i.e. where transhydrogenase acts as a NADPH driven proton pump. Proton pumping was followed using the fluorescent dye 9-amino-6-chloro-2-methoxyacridine (ACMA) which is quenched upon the build-up of an acidic inside pH gradient (Fig. 3A). The reverse reaction is either started by adding NADPH or NAD^+^ (while the other is present from the beginning), leading to a decrease in ACMA fluorescence. In Fig. 3A, the observed ACMA quench was slightly slower and progressed to a lesser maximal quench when the reaction was started with NADPH instead of NAD^+^, however this was not observed in all measurements. If mutant variant H91A was reconstituted, no ACMA quench was observed, supporting the essential role of the H91 for proton pumping (Fig. 3A).

In many bacteria and mitochondria, the membrane potential is the dominant component of the *pmf* (~ − 120 to − 160 mV) while the pH gradient is typically < 0.5 pH units. This makes the membrane potential generated by respiratory complexes the primary driving force for the endergonic forward reaction under cellular conditions, where the NADPH/NADP^+^ is much larger than the NADH/NAD^+^ ratio. We have therefore mimicked this scenario by co-reconstituting TH with ATP synthase from *E. coli.* ATP synthase has the advantage that it can readily be energized with the non-redox active substrate ATP and shows a high unidirectionality of incorporation into liposomes (F_1_ head located to the outside)^42^. We have confirmed the successful co-reconstitution of the two enzymes by a series of experiments. First, we utilized TH as a redox-driven proton pump to establish a *pmf* (inside positive and acidic, as described above) and followed if the established *pmf* is sufficient to support ATP synthesis by ATP synthase. As seen in Fig. 3B, upon addition of NADPH, continuous ATP synthesis is observed using a luciferin/luciferase-based ATP detection system. No ATP synthesis was observed, if H91A variant was reconstituted alongside ATP synthase.

### ATP hydrolysis driven *pmf* generation and NADPH generation by TH

As stated in the introduction, under cellular conditions, the redox potential of the NADPH/NADP^+^ couple is more negative than the NADH/NAD^+^ couple, and the *pmf* is required to push the reaction towards NADPH generation (forward reaction). If the *pmf* is thereby not constantly supplied, the reaction will halt once it has reached its thermodynamic equilibrium. ATP hydrolysis driven proton pumping could represent such as source of *pmf* generation and was mimicked in the following experiment. NADPH and APAD^+^ (an NAD^+^ analogue and substrate of TH) were added to proteoliposomes containing both enzymes and the formation of APADH was followed spectrophotometrically at 375 nm. After a few minutes, the increase in absorption stopped indicating that the reaction reached its chemical equilibrium, i.e. rates of the forward and reverse reactions are identical due to the relative substrate and product concentrations and the established *pmf*. At this point, addition of ATP energized inward proton pumping by ATP synthase, increasing the inside positive membrane potential. Consequently, the reaction moved away from equilibrium, catalysing the forward reaction of TH and thus NADPH formation and APADH consumption which can be observed as a decrease in absorption at 375 nm (Fig. 3C).

### NADH-driven Δψ generation by TH

**Figure 4:**
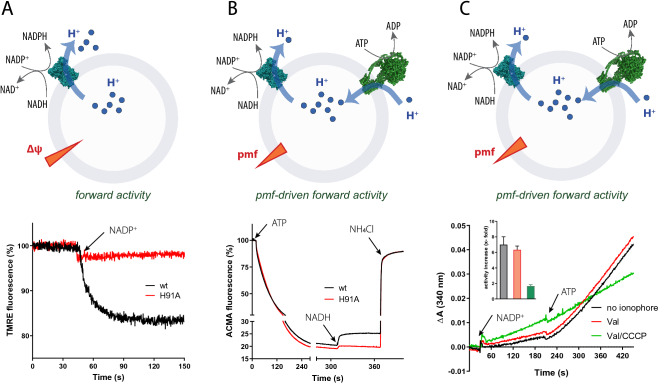
Forward activity of EcTH in reconstituted systems. (**A**) Proton pumping of reconstituted transhydrogenase during forward reaction was measured following TMRE fluorescence at λ_ex/em_ 549/573 nm in 1.5 ml 50 mM HEPES pH 7.5, 100 mM KCl, supplied with 50 μl transhydrogenase proteoliposomes, 4 U/ml glutathione reductase, 1 mM oxidized glutathione, 0.2 μM TMRE, and 200 μM NADH. Upon addition of 800 μM NADP^+^, TMRE fluorescence decreased and a stable plateau was reached. (**B**) ACMA dequench experiment to determine coupling of the co-reconstituted ATP synthase and transhydrogenase proteoliposomes was carried out in 1.5 ml 50 mM HEPES, pH 7.5, 100 mM KCl, 100 ul liposomes, 0.2 µM valinomycin, 4 mU/ml glutathione reductase, 1 mM oxidized glutathione, 2 µM ACMA, 500 µM NADP^+^, and 2 mM MgCl_2_. Upon addition of 250 μM ATP, the *E. coli* ATP synthase establishes a positive inside proton gradient leading to a decrease in ACMA fluorescence. Addition of 500 µM NADH turns on transhydrogenase in the forward direction causing it to pump protons out of the proteoliposomes and leading to a dequench of ACMA fluorescence. Injection of 33 mM NH_4_Cl dissipates the proton gradient re-establishing ACMA fluorescence. (**C**) The acceleration of the transhydrogenase forward reaction in the presence of a membrane potential was measured using co-reconstituted ATP synthase and transhydrogenase proteoliposomes in 50 mM HEPES, pH 7.5, 100 mM KCl, 100 ul liposomes containing 1.2 U/ml alcohol dehydrogenase, 130 mM ethanol, 6 mM hydrazine (pH 7.0), 100 µl liposomes, 0.2 µM valinomycin, 4 mM MgCl_2_, and 200 µM NADH. The reaction was monitored following absorbance of the substrate appearance of NADPH at 340 nm. Forward activity was started by adding 500 µM NADP^+^ leading to an increase in absorbance at 340 nm. Addition of 250 µM ATP, leading to the ATP synthase pumping protons into the vesicle, stimulated NADPH formation rate. Cartoon schemes were drawn in Adobe Illustrator 2021 and graphs created in Prism software (GraphPad, v7.0).

Next, we designed experiments to monitor the forward activity of TH in proteoliposomes under different conditions. Using suitable substrate concentrations that shift the equilibrium of the reaction (high NADH and NADP^+^, low NADPH and NAD^+^), the transhydrogenase can be run in the forward direction. Since only proteoliposomes with TH enzymes having their nucleotide binding domains on the outside will be activated, addition of NADH and NADP^+^ will result in proton ejection from the proteoliposomes. Forward activity is therefore expected to establish a positive outside membrane potential, which can be monitored using the fluorescent potential-sensitive dye tetramethylrhodamine ethyl ester (TMRE). As depicted in Fig. 4A, the generation of a membrane potential is observed as a decrease in TMRE fluorescence. No signal shift was observed with H91A.

### TH forward activity driven by an ATP-driven proton pump

To mimic a more natural situation, a proton pump such as the ATP synthase can be used as a constant *pmf* generator that energizes forward activity of the transhydrogenase enzyme. In the following, two different experimental setups using proteoliposomes containing both TH and ATP synthase from *E. coli* have been used to follow forward TH activity. In the first, proton pumping by either enzyme was detected by ACMA fluorescence. First, ATP synthase was run as a proton pump, establishing an inside acidic pH gradient observed as a quench in ACMA fluorescence (See Fig. 4B). Addition of valinomycin in the presence of potassium ensured that no opposing membrane potential was established during proton pumping. Addition of the substrates NADH and NADP^+^ drives TH in the forward direction which is accompanied by extrusion of protons from the lumen that is in turn observed as an increase in ACMA fluorescence (dequenching) (Fig. 4B). As both systems are continuously working, a new equilibrium is established.

In the second setup, we have reproduced earlier findings^19,22,44^ that demonstrate the direct effect of a *pmf* on the rate of NADPH formation during the forward reaction. The experiments were started by addition of NADH and NADP^+^ either in the absence and presence of valinomycin, as well as in presence of valinomycin and CCCP. In all cases, a continuous production of NADPH was observed (Fig. 4C). Initial NADPH production was fastest in proteoliposomes with supplied valinomycin and CCCP (no opposing *pmf* was generated), whereas liposomes having no ionophores or valinomycin alone showed both lower but very similar NADPH production rates (opposing *pmf* generated in form of a ΔpH which is negative and alkaline inside). Addition of ATP triggered proton influx into the liposomes by the ATP synthase. This established a supportive *pmf* (acidic and positive inside) in form of a ∆Ψ and ∆pH in the proteoliposomes without ionophores, whereas in the proteoliposomes supplied with valinomycin the *pmf* was established in form of a ∆pH alone. In the proteoliposomes supplied with valinomycin and CCCP, addition of ATP is not expected to have a significant effect since the formation of a ∆Ψ and/or a ∆pH is prevented. Addition of ATP increased the rate of NADPH synthesis 7 ± 1.8-fold in the sample without any ionophore and 6.4 ± 0.8-fold in the sample with added valinomycin (Fig. 4C). In presence of both valinomycin and CCCP the stimulation of the forward reaction was essentially lost (1.6 ± 0.3-fold) (Fig. 4C).

## Discussion

Here, we describe the efficient overexpression and Ni–NTA affinity-based purification of the *E. coli* transhydrogenase. The enzyme is obtained in good yields and shows a specific activity of around 6 µmol/mg/min. We have determined the K_M_ values for its natural substrates under similar conditions in the reverse and forward direction, which to our knowledge, has not been described so far. The values are in the same range as previously published values for the bovine enzyme^18^ and transhydrogenase from *Rhodobacter capsulatus*^45^, reflecting the high conservation of the enzyme mechanism. For the *E. coli* enzyme, Meuller et al. investigated the K_M_ values for the reverse reaction using NADPH and the NAD^+^ analogue APAD^+^ as well as for the forward reaction using NADH and thio-NADP^+^^46^. They obtained apparent K_M_ values in the forward reaction of 2 μM and 8 μM for NADH and NADP^+^, respectively. For the reverse reaction, they measured K_M_ values of 20–26 μM and 15 μM for NAD^+^ and NADPH, respectively. In another report, Yamaguchi et al. measured K_M_ values of 33 μM and 54 μM for NADPH and NAD^+^ respectively in the reverse reaction^47^. In general, the found values deviate from each other considerably, but they typically agree on the observation that the K_M_ is lower for the reduced species. Direct comparison of such values is difficult, as the experimental conditions among reports vary significantly, e.g. purified enzyme or membrane vesicles, use of nucleotide analogues or regenerative systems which are required to allow the use of the natural substrates^47^. In summary, the high yield and specific activity as well as the sensible range of K_M_ values obtained with our preparation makes it a suitable expression and purification system for functional (and structural) investigations. Most importantly, it is critical to better understand the functional coupling between the hydride transfer in the soluble dI/dIII interface and the proton transfer through the membrane embedded dII domain. These experiments require the presence of a lipid environment, and our protocols for efficient reconstitution of TH (and ATP synthase) facilitate this critical step. Our measurements showed that a majority (75–85%) of the enzymes is oriented with its large soluble nucleotide binding domains towards the outside. Given the method used for reconstitution, destabilization of preformed liposomes using a defined detergent concentration, this preferred orientation is not surprising^43^. Using TMRE and ACMA fluorescence we show that transhydrogenase establishes a *pmf* by translocating protons across the membrane, both when run in the forward and reverse direction. The establishment of a positive inside proton gradient in proteoliposomes during the reverse reaction has been shown before for the bovine enzyme^17,19,21,48^ as well as for the *E. coli* enzyme^21,22,31,32,46,47,49,50^. We now show that the *pmf* generated during the reverse reaction is sufficient to sustain ATP synthesis by ATP synthase co-reconstituted in the same liposomes. In our experiments, we have used rather high numbers of enzymes per liposomes, yielding only liposomes containing several copies of both types of proteins. The use of non-membrane permeable substrates ensures that only one type of proteoliposomes is functionally relevant. In those, the relative orientation of TH and ATP synthase (both nucleotide binding sites on the same side of the membrane) also reflects the physiological situation. An interesting observation is the almost immediate start of ATP synthesis after NADPH addition. This is noteworthy as compared to typical respiratory enzymes such as complex IV (300 to 1000 H^+^/s), the turnover rate of TH is rather slow (90 ± 17 H^+^/s). The lack of an initial delay indicates that the requirement of ATP synthesis (*pmf* > 120 mV and an inside pH < 6.5) is met very quickly (much quicker than the corresponding ACMA signal), supporting the idea of a local distribution of the pumped protons along the membrane surface^51,52^. Co-reconstitution of TH and ATP synthase from bovine mitochondria was described already earlier by Rydström et al.^18^. According to them, despite repeated attempts they were not able to observe ATP synthesis driven by NADPH driven proton pumping and potential issues are discussed^8^. Our data thus fill this important gap, and demonstrates that TH could potentially act as a *pmf* generator sufficient to energize ATP synthesis in the absence of other suitable reducing equivalents. Using TMRE fluorescence, we also show the formation of a positive outside membrane potential during the forward reaction by reconstituted transhydrogenase using the appropriate substrate concentrations, reinforcing the view that hydride transfer is coupled to proton transport also under the most favourable conditions e.g. nucleotide concentrations.

Our experimental system also allows to observe the stimulating effect of a membrane potential by ATP synthase on transhydrogenase forward activity that has been demonstrated before for the reconstituted bovine enzymes. We find a stimulating rate of approximately 7 in absence of valinomycin which is lower than that found for the reconstituted bovine enzymes by Eytan et al.^19^ but similar to the one found for the reconstituted bovine enzymes by Jolla et al.^21^. Such experiments are important for the investigation of mutant variants that affect the coupling between the two spatially separated reactions. Thanks to the new enzyme structures, more refined mechanistic models have been postulated that now must be biochemically validated. Yet another question, e.g. which step of the proton transport (loading and/or unloading of the proton loading site) is affected by which component of the *pmf* has not yet been discussed or approached at all. Here, we experimentally show that the direction of the reaction can be switched from reverse to forward by applying a *pmf*, once the reaction has reached equilibrium. This finding supports the idea that under physiologic conditions, when the relevant nucleotide concentration are rather constant, transhydrogenase direction and activity is mainly controlled by the presence and extent of the *pmf*. This experimental setup should be useful to assess mutant variants that have an altered proton pathway.

Finally, we present an experiment in which TH forward activity was assessed using ACMA fluorescence. This setup has the unique advantage that both reaction directions can be observed using the natural substrates and followed via transmembrane proton transport, while otherwise nucleotide analogues such as APAD were necessary. In co-reconstituted liposomes, ATP synthase first pumps protons into the lumen which is seen as a large quench in ACMA fluorescence. Addition of NADH and NADP^+^ enables TH to catalyse the forward reaction which is coupled to proton translocation to the outside, seen as a dequench in ACMA fluorescence. It should be noted that in this experiment (Fig. 4B), the rate and the level of *pmf* established by the ATP synthase are identical with either co-reconstituted wt or the non-pumping βH91A mutant. The latter lacks the key histidine and has an interrupted proton translocation pathway that prohibits transmembrane proton transfer^24,26^. Only upon addition of substrates for transhydrogenase forward activity, a change in the signal is observed for the wt enzyme, implying that the transhydrogenase proton translocation channel is gated (by the nucleotides binding sites) to prevent short circuiting of protons and thus loss of *pmf*. In other words, there are no substantial leaks in the proton channel of wt transhydrogenase when it is not activated e.g. in absence of all the required substrates.

## Concluding remarks

In summary, we used established as well as new techniques to monitor transhydrogenase activity, including proton translocation as well as dependence on a membrane potential. The described protocols are straightforward allowing fast handling of this rather delicate enzyme and should provide a good basis for biochemical, biophysical and structural experiments. The different experimental setups provide a broad toolset to investigate different functional aspects of potentially interesting transhydrogenase mutant variants. Liposome experiments are crucial for the investigation of the capability of transhydrogenase to establish and maintain a membrane potential as well as the sensibility towards the presence of a membrane potential when certain key residues are altered. An interesting experiment in this perspective would be to investigate the sensitivity towards the presence of a membrane potential of transhydrogenase mutant variants which have been shown to have impaired activity and/or proton pumping capability, such as mutations of residue αY226 in *E. coli* which is part of the NAD(H) binding pocket^53^ or the critical histidine (βH91) which lies in the proton translocation channel^26,27,54^. Another intriguing setup would be to coreconstitute the membrane portion of transhydrogenase alone and together with ATP synthase. This would allow to investigate whether the channel is open or closed when the soluble domains are absent. Despite the large set of functional data and the recent protein structures, intimate details of this unique molecular mechanism are still to be discovered.

## Supplementary Information

Supplementary Information.
