# Supplementary material for: Energy transfer between the nicotinamide nucleotide transhydrogenase and ATP synthase of Escherichia coli
Source: Sci Rep. 2021 Oct 27;11:21234. doi: 10.1038/s41598-021-00651-6 (PMC8551311; doi:10.1038/s41598-021-00651-6)
Supplement: Supplementary file 1 — Supplementary Information. [file 41598_2021_651_MOESM1_ESM.docx]

# Supplementary Information

# Energy transfer between the nicotinamide nucleotide transhydrogenase and ATP synthase of *Escherichia coli.*

## Simone Sandra Graf^1^, Sangjin Hong^2^, Philipp Müller^1^, Robert Gennis^2^ and Christoph von Ballmoos^1,*^

^1^Department of Chemistry, Biochemistry & Pharmaceutical Sciences, University of Bern, Freiestrasse 3, 3012 Bern, Switzerland

^2^Department of Biochemistry, University of Illinois at Urbana-Champaign, Urbana, Illinois 61801, United States

Correspondence to: Christoph von Ballmoos

Departement für Chemie und Biochemie

Freiestrasse 3

3012 Bern

+41 31 631 43 67

[christoph.vonballmoos@dcb.unibe.ch](mailto:christoph.vonballmoos@dcb.unibe.ch)


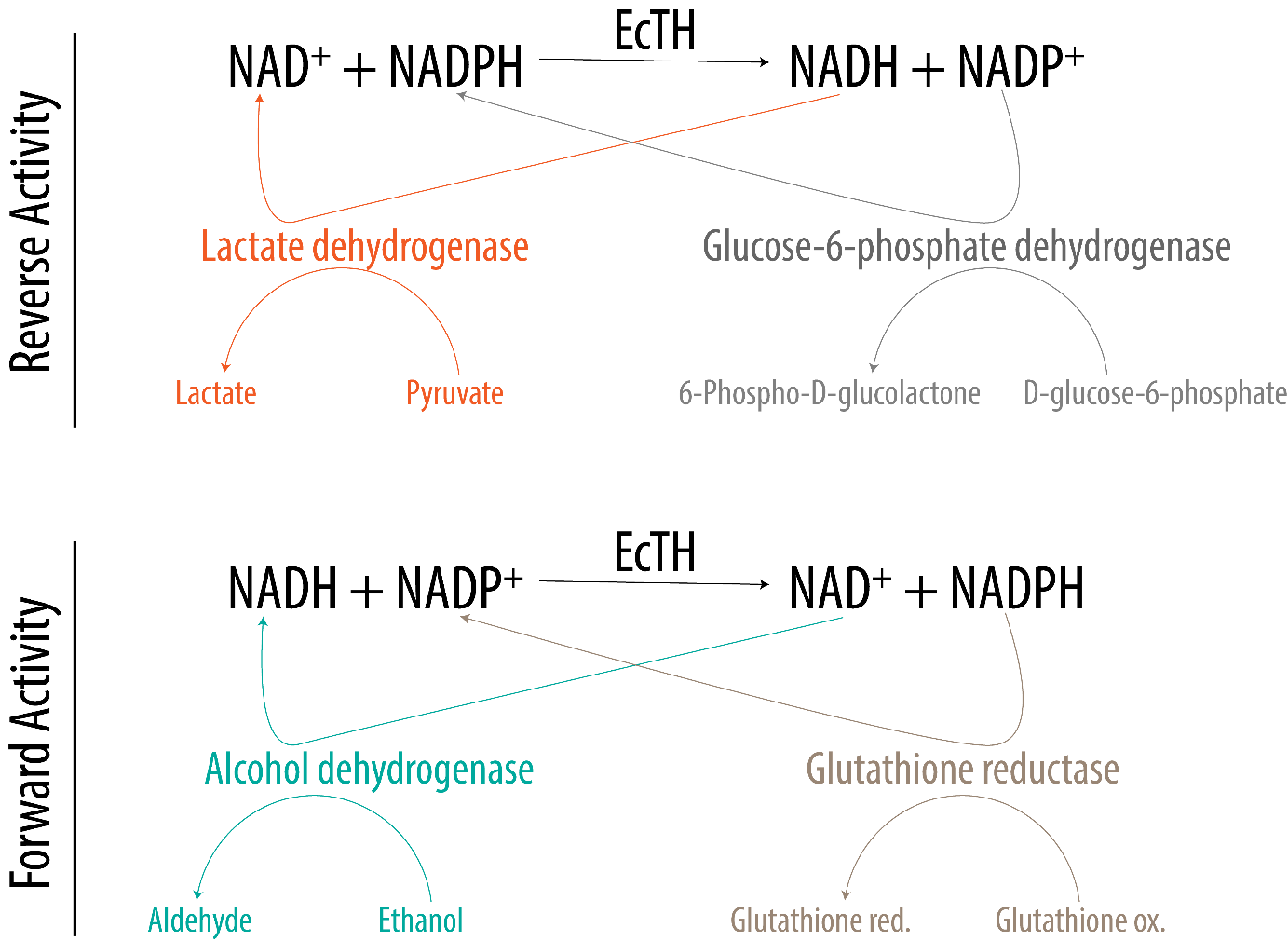


**Figure S1: Scheme displaying enzymes used for substrate regeneration.**

Overview of the regenerating enzymatic systems used for the determination of enzyme activity. For details, see text and Materials and Methods section. Scheme created in Microsoft PowerPoint 2017.


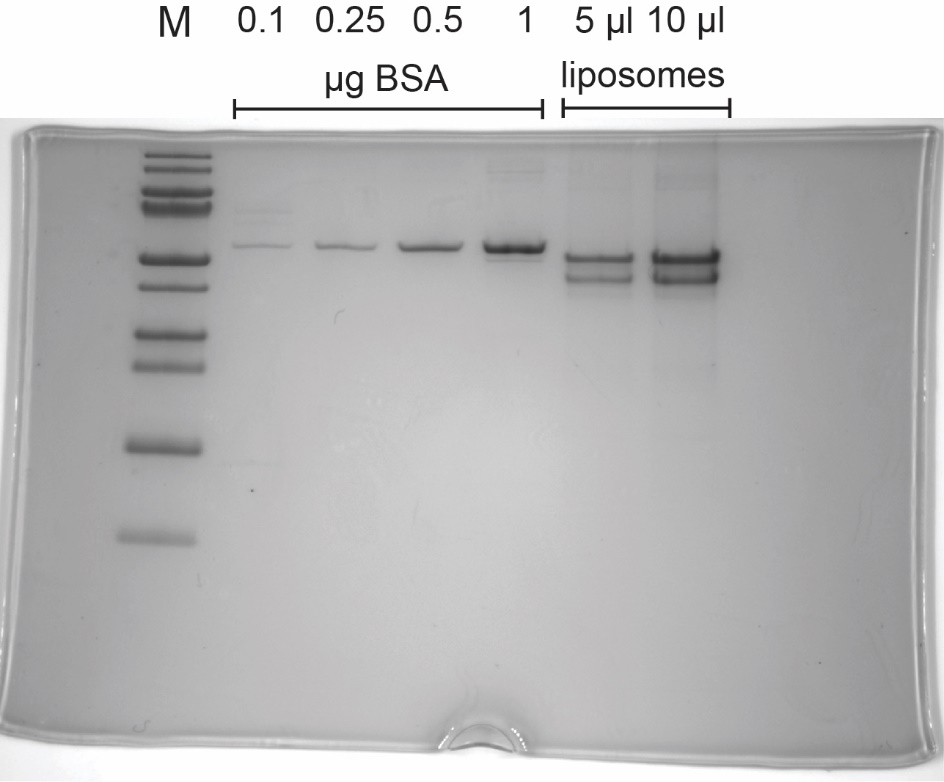


**Figure S2: Analysis of protein reconstitution into liposomes by SDS-PAGE.**

Lanes 1-4 show BSA as a marker for protein content. Lanes 5&6 are 5 and 10 μl of liposomes solubilized in SDS buffer and subjected to PAGE. Gel was scanned, then imported and labeled in Adobe Illustrator 2021.


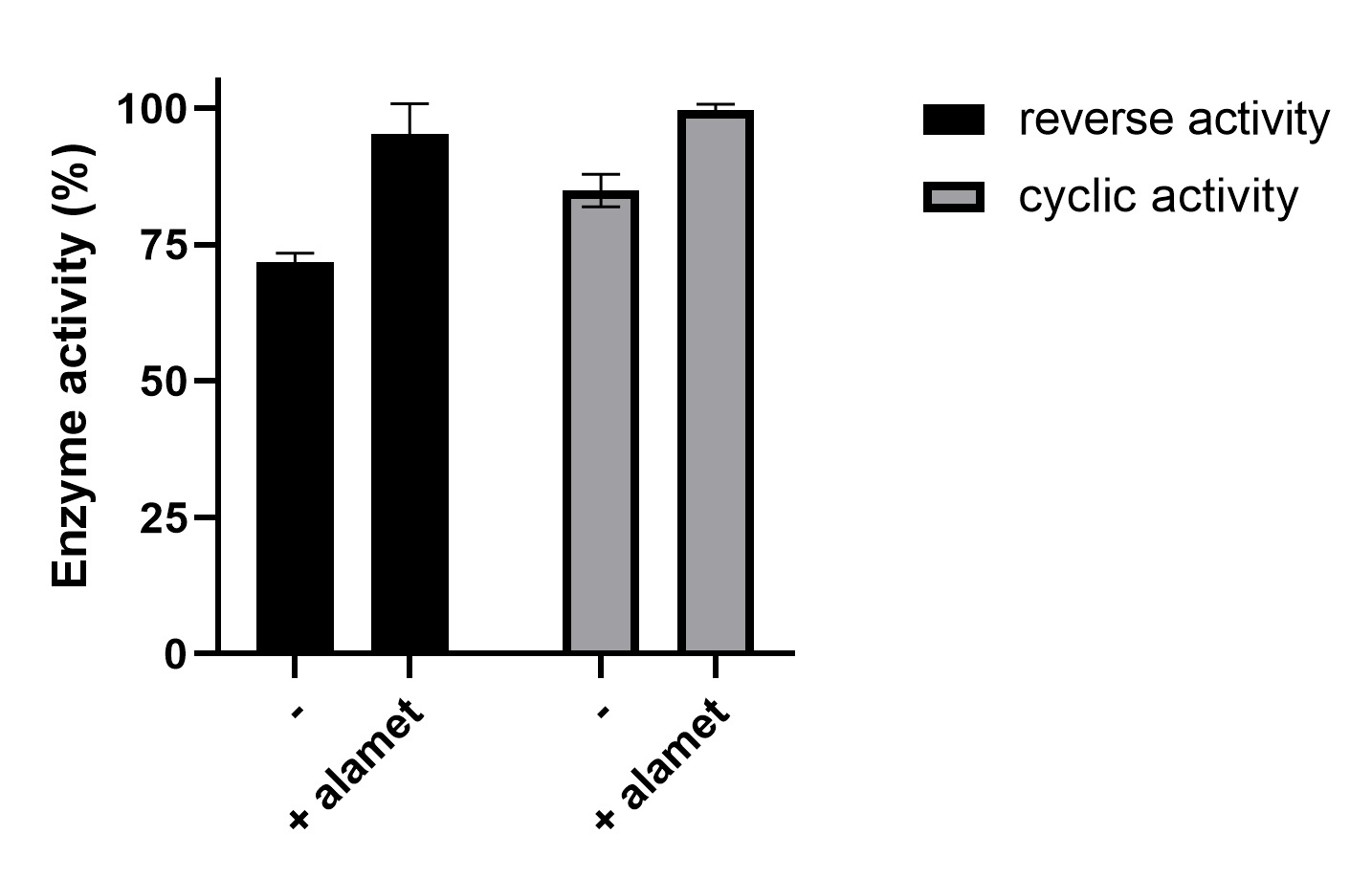


**Figure S3: Determination of enzyme orientation after reconstitution into liposomes.**

Reverse and cyclic enzyme activity was measured in the absence and the presence of the membrane-pore-forming peptide alamethicin. Protein orientation was calculated as ratio between activities in the absence and presence of alamethicin and were found to be between 70% and 85% with the nucleoside binding sites oriented towards the outside (accessible in the absence of alamethicin). For details, see text and Materials and Methods. Graph created in Prism software (GraphPad, v7.0).
